# Application of FRP Bolts in Monitoring the Internal Force of the Rocks Surrounding a Mine-Shield Tunnel

**DOI:** 10.3390/s18092763

**Published:** 2018-08-22

**Authors:** Zhen Liu, Cuiying Zhou, Yiqi Lu, Xu Yang, Yanhao Liang, Lihai Zhang

**Affiliations:** 1 School of Civil Engineering, Sun Yat-sen University, No. 135 XinGangXiLu, Guangzhou 510275, China; liuzh8@mail.sysu.edu.cn (Z.L.); luyiqi3@mail.sysu.edu.cn (Y.L.); yangxu9@mail.sysu.edu.cn (X.Y.); liangyh33@mail2.sysu.edu.cn (Y.L.); 2 Guangdong Engineering Research Centre for Major Infrastructure Safety, School of Civil Engineering, Sun Yat-sen University, Guangzhou 510275, China; 3 Research Center for Geotechnical Engineering and Information Technology, Sun Yat-sen University, No. 135 XinGangXiLu, Guangzhou 510275, China; 4 Department of Infrastructure Engineering, The University of Melbourne, Melbourne VIC 3010, Australia; lihzhang@unimelb.edu.au

**Keywords:** fiberglass reinforced plastic bolt, monitoring, mine-shield tunnel, internal force of rocks, corrosive environment

## Abstract

Monitoring the internal force of the rocks surrounding a mine-shield tunnel for the initial support of a mine-shield tunnel, in complex geological and hydrological environments, requires bolts with specific features such as high tensile strength, low shear strength, good insulation and resistance to corrosion. As such, internal force monitoring has become an important issue in safety monitoring for such tunneling projects. In this paper, the adaptability of a mine-shield tunnel project in a corrosive environment is investigated. A fiberglass reinforced plastic (FRP) bolt with high tensile strength, low shear strength, resistance to fatigue, non-conductivity and resistance to corrosion is used as a probe in tandem with an anchor-head dynamometer to monitor the internal force of the rocks surrounding a mine-shield tunnel for initial support. Additionally, solar energy collection technology is introduced to create a remote monitoring system. Using a 2.5 km long railway tunnel located in the northeast of the Pearl River Delta of China as a case study, the present study shows that, compared with a conventional steel bolt, the FRP bolt has advantages, such as avoidance of the risks associated with the shield machine, insulation and resistance to corrosion. As a probe, the response of the FRP bolt to events such as a blasting vibration and a construction disturbance that results in internal changes in the surrounding rock demonstrates a clear pattern that is appropriate for monitoring the internal force of the rocks surrounding a mine-shield tunnel in a corrosive environment. FRP bolt-based monitoring not only provides new technological support for controlling the risk involved in the initial support of a mine-shield tunnel but can also be widely deployed in projects with special requirements for disassembly, conductivity and corrosion.

## 1. Introduction

Tunnel construction in complex geological and hydrological environments involve a combination of different construction methods to ensure both the safety and on-schedule progress of construction. As such, safety monitoring and feedback between different construction methods is critical [1,2]. Among them, due to its superior feasibility and reliability, the initial mine-tunneling and subsequent shield segment concatenation construction method is widely adopted for tunnel construction in composite formations with inconsistent hardness. Therefore, monitoring the internal force of the rocks surrounding a mine-shield tunnel for initial support after mine tunnel construction, and before shield segment concatenation, has become an important component of feedback-based control of the overall tunnel construction process.

Monitoring the internal force of the rocks surrounding a mine-shield tunnel is primarily based on the bolt-force measurement method. This study focuses on mechanical information collection [3,4], which includes electrical sensor technology and optical sensor technology. Electrical sensor technology is relatively old. An electrical sensor is attached to the bolt’s anchor to convert mechanical vibrations into electrical signals for identification; examples include Lamb wave technology [5] and piezoelectricity-based monitoring technologies [6] such as electromechanical impedance technology (EMI) [7,8]. Optical sensor technology is still under development and is primarily divided into two categories; one deploys optical sensors along bolts to monitor stress variations in the bolt’s main stress node, e.g., evaluating the bolt’s stress and strain distributions via distributed optical sensors [9,10]; the other converts an anchor-head electrical sensor to an optical sensor and operates on a principle similar to that of an electrical sensor, e.g., monitoring via an embedded optical sensor at the anchor head [11,12]. However, the aforementioned technologies ignore the adaptability of the probe bolt to the project and primarily employ steel bolts [13,14]. In particular, monitoring the internal force of the rocks surrounding a mine-shield tunnel for initial support via the mine-shield method in a complex geological and hydrological environment requires bolts with specific features such as high tensile strength, low shear strength, good insulation and resistance to corrosion, which is an issue that needs investigated and addressed in large-scale underground project construction.

Considering this, the adaptability to tunnel projects via the mine-shield method in a corrosive environment is investigated in this paper. A fiberglass reinforced plastic (FRP) bolt with high tensile strength, low shear strength, resistance to fatigue, non-conductivity and resistance to corrosion [15,16] is used as probe in tandem with an anchor-head dynamometer to monitor the internal force of the rocks surrounding a mine-shield tunnel for initial support. Additionally, solar energy collection technology is introduced to create a remote monitoring system. This satisfies the specific requirements of a mine-shield tunnel project in a corrosive environment, such as disassembly, conductivity and corrosion [17]. Although a steel probe bolt has relatively high tensile strength, it can easily damage the cutter head of a shield machine. In addition, the conductivity and corrosion characteristics of a steel bolt may incur serious safety risks if the monitoring work is conducted in a complex environment (e.g., thunderstorms). In contrast, the FRP bolts presented in this study can potentially overcome the above-mentioned shortcomings of conventional steel bolts and therefore mitigate the risks during the monitoring processes. Furthermore, after the completion of tunnel excavation, the cutter head of the shield machine can easily cut the FRP bolts as the shield progresses without damaging the cutter head due to the relatively low shear strength of FRP bolts.

## 2. Research Method

### 2.1. Project Overview

**Figure 1:**
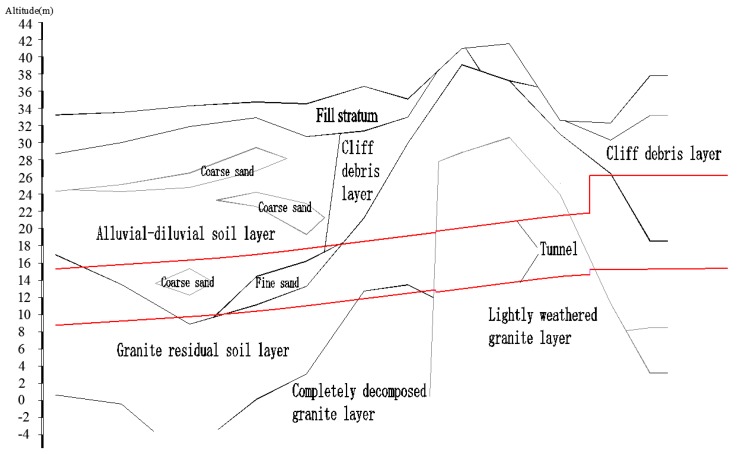
Vertical geological section of the tunnel project.

The project investigated in this study is a tunnel project in an east-west rail transport zone. It is located northeast of the Pearl River Delta in China. The total length is approximately 2.5 km; the shape is circular with a radius of approximately 3.35 m and an average burial depth of approximately 18 m. The tunnel traverses formations that primarily consist of lightly to fully weathered granite, a plastic-hard granite residual soil layer (Figure 1). The environment is slightly acidic with high concentrations of ammonia and nitrogen.

**Figure 2:**
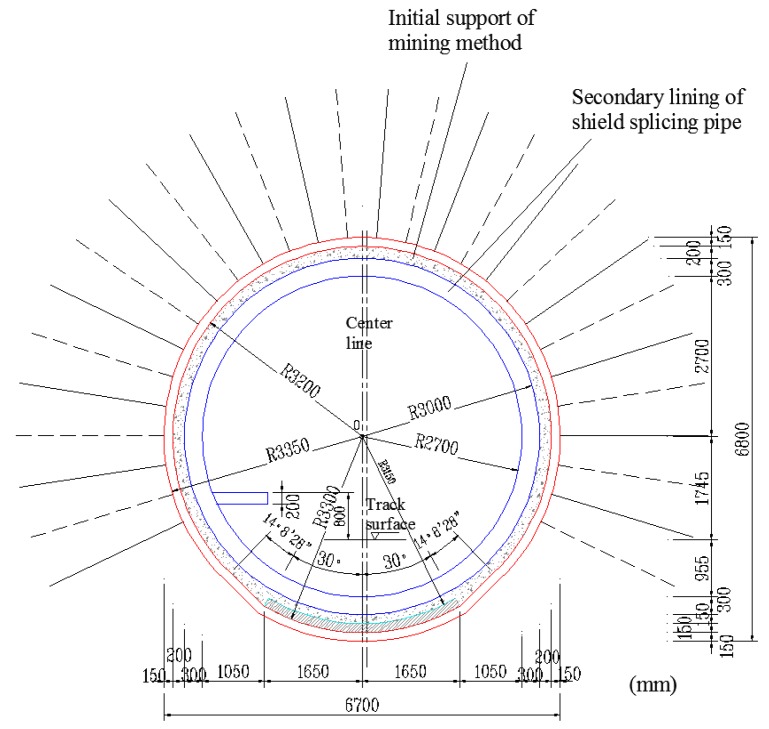
Transverse geological section of the tunnel project.

The tunnel is constructed via the shield method from west to east. In the 100-m area around the East terminal, the tunnel traverses lightly weathered granite and boulders with a rising local rock surface, which creates challenges for shield drilling. Therefore, in the 100-m area in the east, construction is based on initial mine-tunneling excavation followed by shield segment concatenation (Figure 2). This consists of initial support for the outer layer provided by bolts, a rebar network, a steel frame and shotcrete and a secondary inner lining layer made of reinforced concrete segments. The mine-tunneling method is based on multi-step excavation. For lightly weathered granite and boulders with solid surrounding rocks, excavation is based on controlled blasting technology.

According to the safety requirements for underground track transport in tunnels, the internal force of the surrounding rocks should be monitored during mine tunnel construction to perform on-demand adjustments of the design parameters and construction method and to ensure the safety and cost-effectiveness of the tunnel structure.

### 2.2. Principle of Bolt-Based Monitoring of the Internal Force of the Surrounding Rocks for Initial Support

Monitoring the internal force of the rocks surrounding a mine-shield tunnel for initial support is usually based on a bolt dynamometer. As the conventional bolt + anchor-head dynamometer is less sensitive to variations in the internal force of the surrounding rocks, a bolt with a smaller diameter than a reinforcing bolt and micro-tension is normally used as a probe for monitoring. However, the secondary lining of the support project consists of concatenated shield segments. Therefore, bolt-based monitoring of the internal force of the surrounding rocks is only feasible for initial support via the mine-tunneling method. When the shield progresses, it cuts the bolt. A conventional steel bolt is likely to induce accidents such as shield screw conveyors, cutter heads and disc cutter blockages, and is therefore extremely risky. Both shield machine propulsion and vehicle tunnel traversal during operation could generate powerful currents, and the conductivity of a conventional steel bolt could also cause accidents. Furthermore, as the groundwater is slightly acidic with high concentrations of ammonia and nitrogen it is highly corrosive to the bolt. Therefore, bolt-based monitoring of the internal force of the rocks surrounding a mine-shield tunnel in the support project should satisfy requirements such as tensile resistance, ease of shearing, insulation and resistance to corrosion. A FRP bolt has properties such as high tensile strength, low shear strength, resistance to fatigue, non-conductivity and resistance to corrosion and can meet all of the aforementioned requirements. In view of this, a FRP bolt is selected as a probe to monitor variations in the internal force of the surrounding rocks for initial support in tandem with an anchor-head dynamometer.

**Figure 3:**
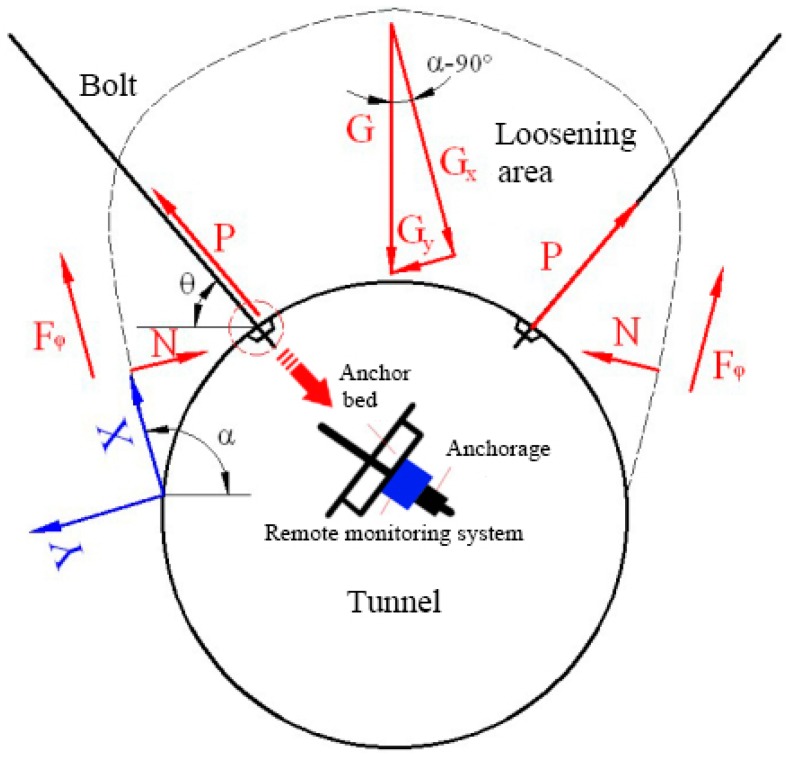
The principle of bolt-based monitoring of the internal force of surrounding rocks for initial support.

Based on the equilibrium of the tunnel’s surrounding rocks under gravity, the relationship between the sliding force of unstable rock soil, the stress on the monitoring bolt and the failure surface friction resistance is created (Figure 3).

The equation relating the sliding force is as follows:

(1) $$G_{t}=P\cdot [\cos(\alpha +\theta )+\sin(\alpha +\theta )\cdot tg\bar{\phi }]+G\cdot \cos\alpha \cdot tg\bar{\phi }+c\cdot l$$

Based on Equation (1), the measured bolt tension is converted to a sliding force.

Based on the principle of a mechanical vibration system, an anchor-head dynamometer can measure the sliding force in rock soil via a FRP bolt.

### 2.3. Equipment for Monitoring the Internal Force of the Surrounding Rocks Using a FRP Bolt for Initial Support

The equipment for monitoring the internal force of the rocks surrounding a mine-shield tunnel for initial support primarily consists of a FRP bolt, a dynamometer, information collection and transmission equipment and energy supply equipment.

(1) FRP bolt

**Figure 4:**
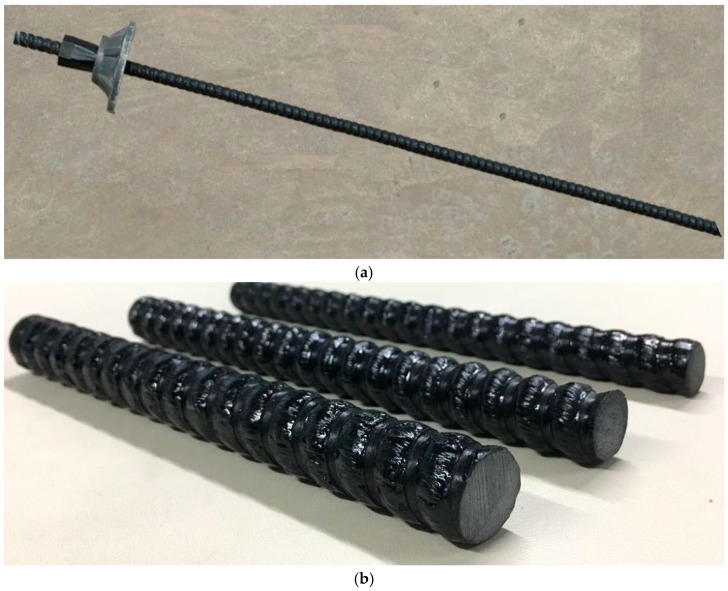
(**a**) A fiberglass reinforced plastic (FRP) bolt; (**b**) detail of the FRP bolt.

**Figure 5:**
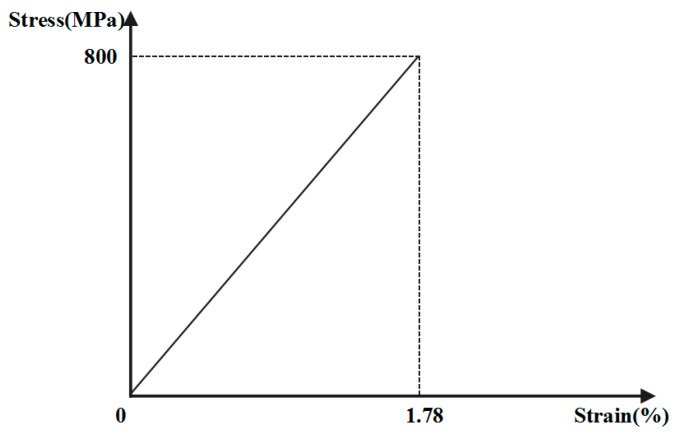
Constitutive relation of the FRP bolt.

The FRP bolt (Figure 4a,b) parameters are as follows: a length of approximately 8.2 m; a diameter of 18 mm; a tensile capacity of 203 kN; a shear capacity of 101 kN and a modulus of elasticity of 45 GPa. The constitutive relation of the FRP bolt is given in Figure 5. The borehole aperture of the bolt is no smaller than 22 mm. A tray structure is used to support the dynamometer.

(2) Dynamometer

**Figure 6:**
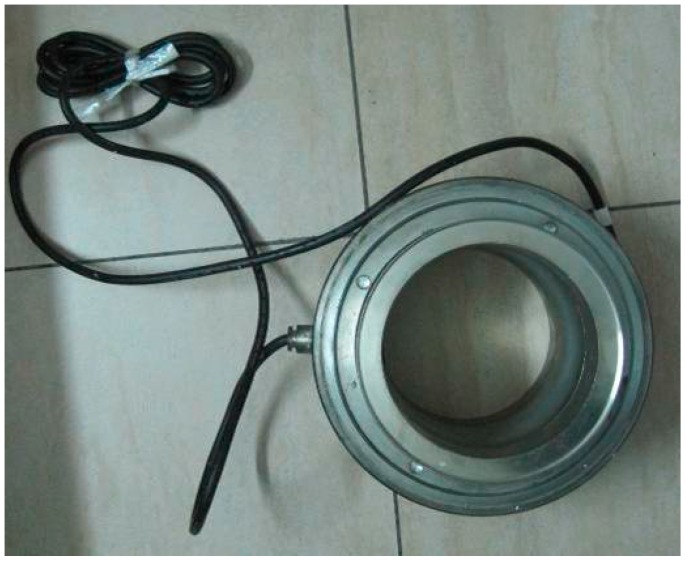
Dynamometer.

The dynamometer (Figure 6) measures variations in the steel string’s vibration frequency to determine the tension in the bolt induced by rock soil sliding. The measurement range is 1800–5000 kN, and the precision is 1 kN.

(3) Information Collection and Transmission Equipment

The information collection and transmission equipment consists of a mechanical information-collecting module, a computing module and a transmission module. The operational temperature is from −30 °C to 50 °C. The mechanical information-collecting module automatically collects the steel string vibration frequencies detected by a high-precision mechanical sensor. The maximum collecting frequency is 5 Hz. The computing module converts the collected vibration frequencies to bolt tension measurements and, further, to binary format by averaging. The information transmission module transmits the collected data to receiving terminals, such as a PC and a mobile phone, via a commercial wireless communication network. The data transmission frequency is 2–5 Hz.

(4) Energy Supply Equipment

The energy supply equipment is based on a solar energy power supply system, which consists of a lithium battery and a solar panel. The solar panel converts solar energy into electrical energy, which is stored in the lithium battery to provide power for the field equipment and to ensure long-term monitoring. The output voltage is 3.7 V, and the operational temperature range is from −15 °C to 40 °C.

### 2.4. Deployment of FRP Bolt-Based Monitoring of the Internal Force of the Surrounding Rocks for Initial Support

**Figure 7:**
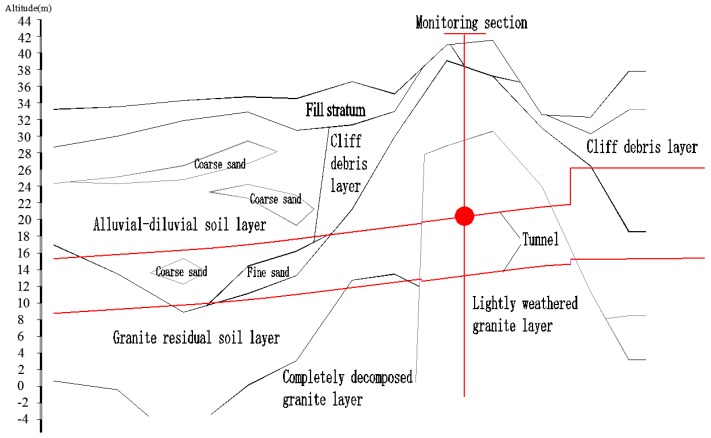
Vertical section of the monitoring point.

Based on the geological conditions of the support project and the requirements of the construction organization, one FRP bolt-based monitoring point is deployed. The milestone number of the monitoring point is ZDK39 + 153 m, and the vertical distance from the surface is 20 m. A vertical section showing the monitoring point is shown in Figure 7.

**Figure 8:**
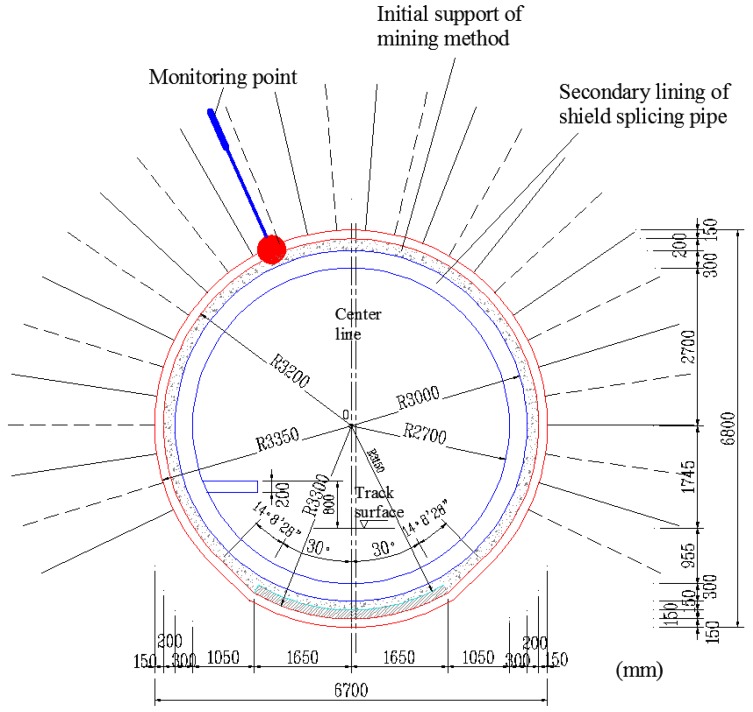
Transverse section showing the monitoring point.

The tunnel section is a circle with a radius of approximately 3.35 m. Excluding the initial support and segment, the tunnel clearance radius is approximately 3 m. Based on the actual operability and the requirement for continuous metro operation without interruption, a monitoring point is located at 330° around the section, as shown in Figure 8. The calculation shows that the length of the FRP bolt’s anchorage segment is 1.5 m and that the free segment length is 3.5 m.

**Figure 9:**
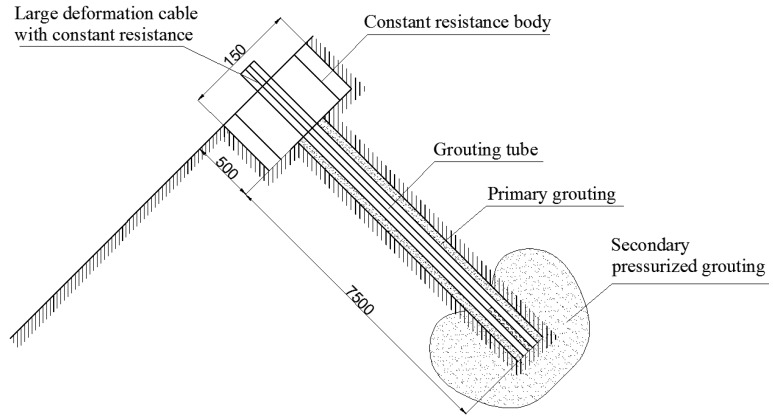
Double pressurized grouting.

Construction of the FRP bolt is based on double grouting. The first is conventional grouting. The second is pressurized grouting (Figure 9) and the grouting pressure is 5–6 MPa.

### 2.5. Remote Monitoring System

Based on the principles of an intelligent data warehouse, a SQL database, an ASP webpage design and flash technology and a remote monitoring and warning system is created. The system consists of three parts: a data-receiving workstation, a central database and a data-processing workstation.

**Figure 10:**
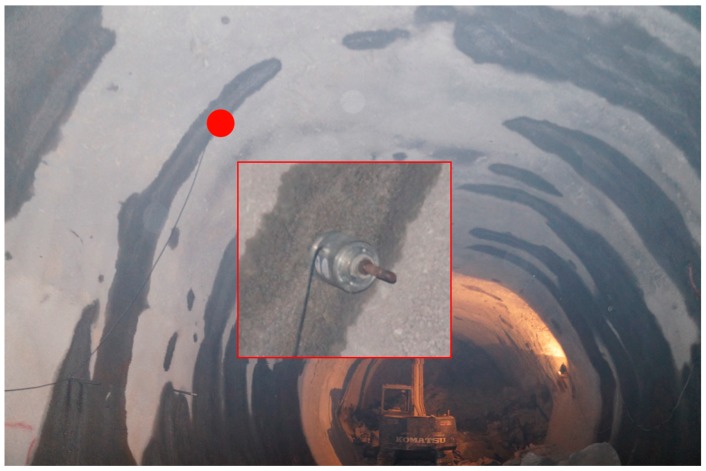
Field deployment of the monitoring point.

Field deployment of the monitoring point is shown in Figure 10. Based on the actual requirements of the support project, the data collection frequency during the monitoring period is set to once per hour.

### 2.6. Disposal of the FRP Bolt during Shield Segment Concatenation

Once tunnel mining excavation is completed, the shield progresses and concatenates segments, based on the initial support, to form a secondary support lining. To ensure smooth progress of the shield machine and quality of the segment concatenation, the dynamometer is removed before the shield machine reaches the monitoring point. After that, the shield machine’s cutter head cuts the FRP bolt. As the FRP bolt has low shear strength and is brittle, it is cut into fragments and has no impact on the shield machine and the segment concatenation.

## 3. Results and Discussion

**Figure 11:**
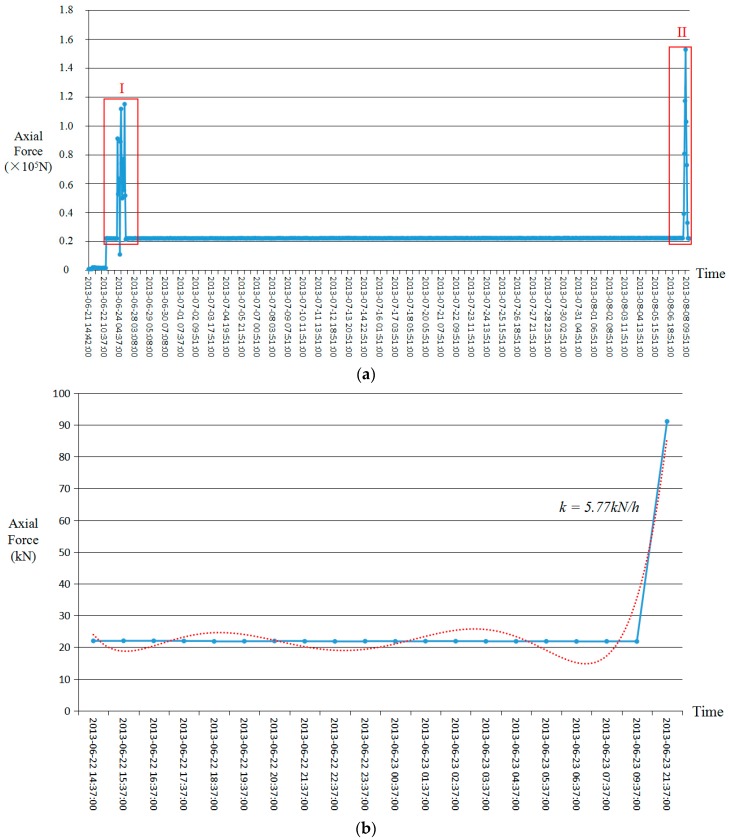

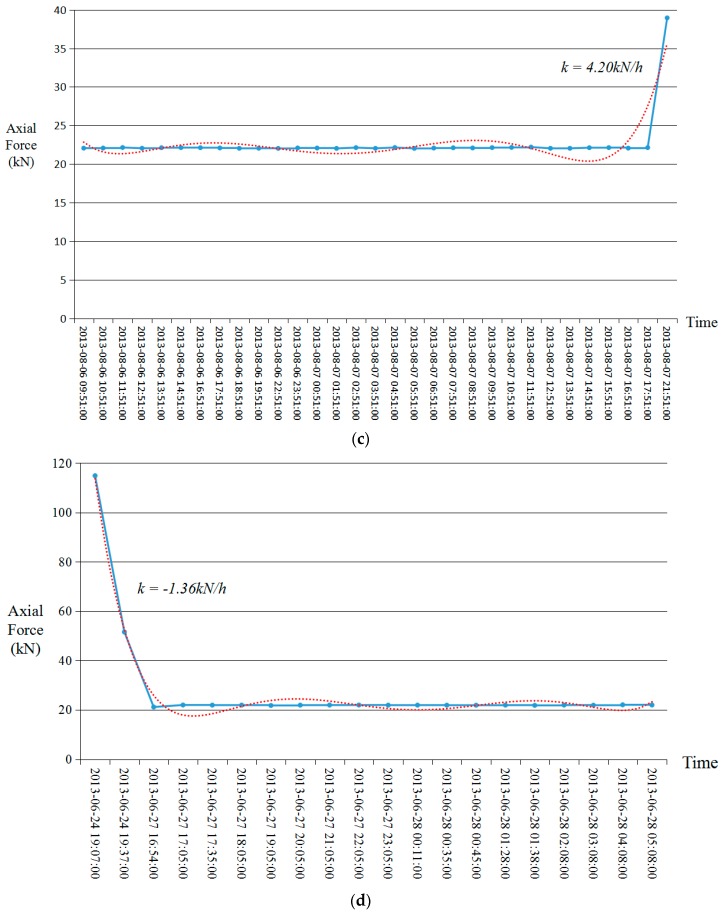
The time-dependent axial force measured by FRP (the *x*-axis is time in year-month-day: hour: minute: second). (**a**) Original monitoring data; (**b**) the rising section of red box I in (**a**); (**c**) the rising section of red box II in (**a**); (**d**) the descending section of red box I in (**a**).

Figure 11a shows a graph of the original monitoring data, which contains 1020 data points. Figure 11b,c shows the rising sections of red box I and red box II in Figure 11a. Figure 11d shows the descending section of red box I in Figure 11a. Based on the monitoring data (Figure 11a), the internal force of the surrounding rocks was in a steady state (about 22 kN) during the period between the initial support and the segment concatenation process. Normal mine-tunneling construction and shield machine progress has had little impact on the internal force of the surrounding rocks. As shown in Figure 11b–d, the increasing rate (4.20–5.77 kN/h) of internal force of the surrounding rocks, due to blasting, is higher than the decreasing rate (1.36 kN/h) after blasting. The decreasing rate of the internal force of surrounding rocks after the final blasting is not considered because the shield machine was advancing to the monitoring point.

**Figure 12:**
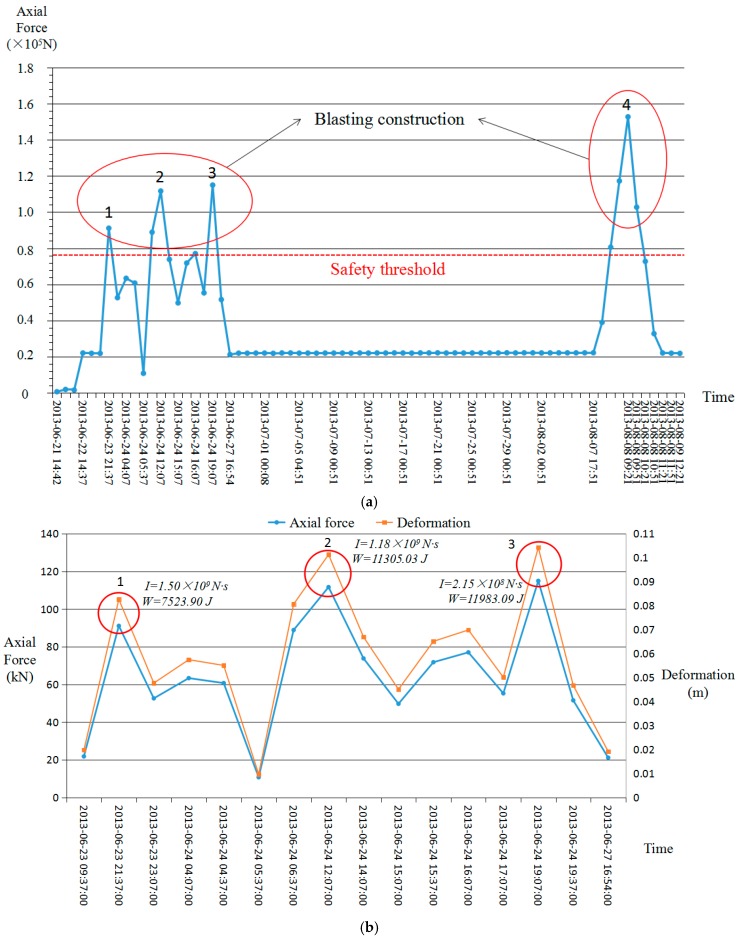

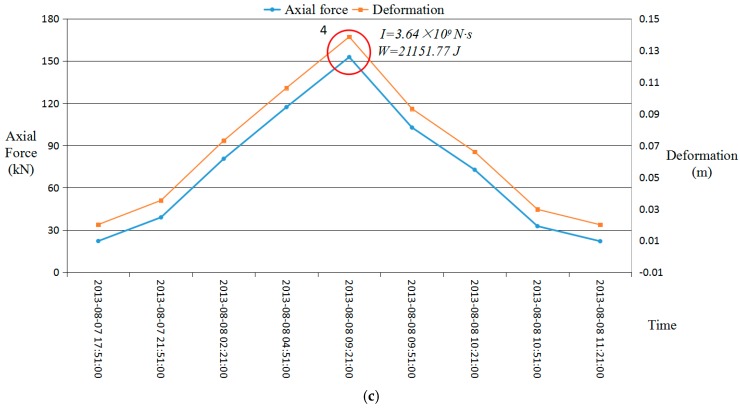
Simplified monitoring data of the time-dependent axial force measured by FRP (the *x*-axis is time in year-month-day: hour: minute: second). (**a**) Simplified monitoring data; (**b**) the data of the left circle in (**a**); (**c**) the data of the right circle in (**a**).

By simplifying the steady state monitoring data points of Figure 11a, we can get Figure 12a. In Figure 12a, the data in the red circles were obtained during construction blasting and the red line is the safety threshold. Figure 12b,c illustrates the data from the red circles in Figure 12a. There were four explosions and the blasting time was 2–20.5 h. Under the influence of blasting, the internal force of surrounding rocks varied greatly. The deformation was caused by the impulse in the monitoring period and the work of FRP bolt is complete. As shown in Figure 12b, three blasts produced impulses, displacements and works on surrounding rocks in 14 h, 6.5 h and 2 h, respectively. The first blasting lasted longer than the following two blasts, so the impulse (1.50 × 10^9^ N·s) was higher. The internal force (114.94 kN) and deformation (0.10 m) of the surrounding rocks due to the third blasting was larger than the first two blasts, so the work (11,983.09 J) was higher. Due to the readjustment of the internal force of surrounding rocks after blasting, the internal force decreased and then rose lightly after the first and second blasting. In Figure 12c, both the impulse (3.64 × 10^9^ N·s) and work (21,151.77 J) of the fourth blasting was higher than the previous three. The highest quantity of explosives was used in the fourth blasting. Thus, the position of the fourth blasting was further from the monitoring point, but the data value was higher than the other three times.

In comparison, blasting has a significant short-term impact on the internal force of the surrounding rocks because the internal force of the surrounding rocks exhibits a clear, abrupt change of 5–7 times the normal range and should be handled properly during project construction (red circles in Figure 12a). This also means that FRP bolt-based monitoring is not only very effective at monitoring tunnel stability, but also has a clear response pattern for events that could result in internal changes in the surrounding rocks such as blasting vibration and construction perturbation. The data collection frequency during the monitoring period is once per hour. The blasting signal could not be collected if the acquisition frequency was too low and if the amount of information increased. Based on the actual requirements of the support project it is also unnecessary if the frequency is too high. Therefore, it is completely suitable for monitoring the internal force of the rocks surrounding a mine-shield tunnel in a corrosive environment.

In this case, the axial force of the FRP bolt can be used as a measure of the safety index according to the threshold standard provided by the engineering company. There is a warning signal when the axial force is greater than 76.36 kN, the strain of the FRP bolt is larger than 0.67% and the data changes suddenly and continuously rises. The evolution of tunnel surrounding rocks can be divided into four modes: stable (the internal force of surrounding rocks is lower than the threshold), sudden disturbance (such as blasting), softening indentation (the internal force of the surrounding rocks is lower than the threshold value with a downward trend) and large deformation (the internal force of the surrounding rocks exceeds the threshold and rises for a long time). According to Figure 12a, the internal force variation pattern of the surrounding rocks was due to the sudden disturbance caused by blasting. After blasting, the internal force of the surrounding rock returned to the stable value. Based on the analysis in combination with engineering practice, the safety of tunnels surrounded by rock is verified.

## 4. Conclusions

In this study, the effectiveness of FRP bolts in monitoring the internal force of the surrounding rocks during tunnel construction is systematically investigated by using a 2.5 km long railway tunnel located in the northeast of the Pearl River Delta of China as a case study. The main conclusions are as follows:

- Compared with a conventional steel bolt, a FRP bolt has several advantages (e.g., high tensile strength, relatively low shear strength, extremely low conductivity, corrosion resistance), and therefore, can mitigate the risks during monitoring processes.
- Through the monitoring of blasting vibration and construction perturbation, the FRP bolt-based system provides an effective and safe method for remotely monitoring the internal mechanical behaviour of rocks surrounding a mine-shield tunnel.

It should be mentioned that the results from this study were mainly dependent on the data obtained from one monitoring point due to the limitation of site conditions. To assess the capability of FRP bolts for monitoring the internal force of the rocks surrounding a mine-shield tunnel, further testing on additional monitoring points is required and is the focus of future study.
